# Effects of acetyl-DL-leucine on cerebellar ataxia (ALCAT trial): study protocol for a multicenter, multinational, randomized, double-blind, placebo-controlled, crossover phase III trial

**DOI:** 10.1186/s12883-016-0786-x

**Published:** 2017-01-10

**Authors:** Katharina Feil, Christine Adrion, Julian Teufel, Sylvia Bösch, Jens Claassen, Ilaria Giordano, Holger Hengel, Heike Jacobi, Thomas Klockgether, Thomas Klopstock, Wolfgang Nachbauer, Ludger Schöls, Claudia Stendel, Ellen Uslar, Bart van de Warrenburg, Ingrid Berger, Ivonne Naumann, Otmar Bayer, Hans-Helge Müller, Ulrich Mansmann, Michael Strupp

**Affiliations:** 1 Department of Neurology with Friedrich-Baur-Institute, University Hospital, Munich, Germany; 2 German Center for Vertigo and Balance Disorders (DSGZ), University Hospital, Munich, Germany; 3 Institute for Medical Informatics, Biometry and Epidemiology (IBE), University Hospital, Munich, Germany; 4 German Center for Neurodegenerative Diseases (DZNE), Munich, 80336 Germany; 5 Department of Neurology, University Hospital Essen, University of Duisburg-Essen, Essen, Germany; 6 Department of Neurology and Hertie Institute for Clinical Brain Research, University Hospital Tübingen, Tübingen, Germany; 7 German Center for Neurodegenerative Diseases (DZNE), Center for Clinical Research, Bonn, Germany; 8 Department of Neurology, Medical University Innsbruck, Innsbruck, Austria; 9 Department of Neurology, Donders Institute for Brain, Cognition, and Behaviour, Radboud University Medical Centre, Nijmegen, Netherlands; 10 Institute for Medical Biometry and Epidemiology, Philipps University Marburg, Marburg, Germany; 11 Department of Neurology and German Center for Vertigo and Balance Disorders, University Hospital, Campus Grosshadern, Marchioninistrasse 15, Munich, 81377 Germany; 12 Munich Cluster for Systems Neurology (SyNergy), Munich, 80336 Germany

**Keywords:** Cerebellar ataxia, Amino acids, Acetyl-DL-leucine, Symptomatic therapy, Randomized controlled trial, Crossover design, Ataxia rating scales, Patient questionnaires, Quality of life, 30.040 Ataxia, 20.060 Clinical trial, 10.070 Movement disorders, 30.260 Neurodegeneration, 20.140 Qualitative research

## Abstract

**Background:**

Cerebellar ataxia (CA) is a frequent and often disabling condition that impairs motor functioning and impacts on quality of life (QoL). No medication has yet been proven effective for the symptomatic or even causative treatment of hereditary or non-hereditary, non-acquired CA. So far, the only treatment recommendation is physiotherapy. Therefore, new therapeutic options are needed. Based on three observational studies, the primary objective of the acetyl-DL-leucine on ataxia (ALCAT) trial is to examine the efficacy and tolerability of a symptomatic therapy with acetyl-DL-leucine compared to placebo on motor function measured by the Scale for the Assessment and Rating of Ataxia (SARA) in patients with CA.

**Methods/Design:**

An investigator-initiated, multicenter, European, randomized, double-blind, placebo-controlled, 2-treatment 2-period crossover phase III trial will be carried out. In total, 108 adult patients who meet the clinical criteria of CA of different etiologies (hereditary or non-hereditary, non-acquired) presenting with a SARA total score of at least 3 points will be randomly assigned in a 1:1 ratio to one of two different treatment sequences, either acetyl-DL-leucine (up to 5 g per day) followed by placebo or vice versa. Each sequence consists of two 6-week treatment periods, separated by a 4-week wash-out period. A follow-up examination is scheduled 4 weeks after the end of treatment. The primary efficacy outcome is the absolute change in the SARA total score. Secondary objectives are to demonstrate that acetyl-DL-leucine is effective in improving (1) motor function measured by the Spinocerebellar Ataxia Functional Index (SCAFI) and SARA subscore items and (2) QoL (EuroQoL 5 dimensions and 5 level version, EQ-5D-5 L), depression (Beck Depression Inventory, BDI-II) and fatigue (Fatigue Severity Score, FSS). Furthermore, the incidence of adverse events will be investigated.

**Discussion:**

The results of this trial will inform whether symptomatic treatment with the modified amino-acid acetyl-DL-leucine is a worthy candidate for a new drug therapy to relieve ataxia symptoms and to improve patient care. If superiority of the experimental drug to placebo can be established it will also be re-purposing of an agent that has been previously used for the symptomatic treatment of dizziness.

**Trial registration:**

The trial was prospectively registered at www.clinicaltrialsregister.eu (EudraCT no. 2015–000460–34) and at https://www.germanctr.de (DRKS-ID: DRKS00009733).

## Background

Cerebellar ataxia (CA) is a frequent and often disabling syndrome which can severely impair motor functioning and quality of life (QoL) [1]. CAs are most often caused by neurodegenerative disorders of the cerebellum which are either hereditary or non-hereditary (sporadic); all in all, about 50% of the cases are sporadic. Studies suggest that the overall prevalence of CA in Europe is similar to that in Japan and may approach 20:100,000, i.e., 15,000 patients in Germany (www.ataxie.de) [2]. Due to the various etiologies and different courses of the diseases, there are no robust epidemiological data on CA-related mortality.

The leading clinical symptoms of CA are disturbances of stance or gait (>85%) with recurrent falls, limb ataxia with severe functional impairment of arm and hand movements, dysarthrophonia with impaired oral communication abilities, and ocular motor disturbances with impaired vision [1]. Further, most types of CA are progressive and therefore become more disabling in the course of the disease, severely impairing QoL and functioning [1]. In addition to functional impairment, CA also affects cognitive and psychosocial abilities and limits the ability to perform tasks of daily life. It is thus a severely disabling condition, progressively restricting autonomy and social participation. The care that is required is frequently provided by the family, thus further increasing the socioeconomic burden of disease [3]. Major cost components relevant for patients with CA are informal care, early retirement because of permanent disability, drugs, orthopedic devices, and rehabilitation. As the disease progresses, quality of life decreases and utilization of health resources increases [4].

No medication has yet been proven effective for the symptomatic or even causative treatment of degenerative CA [5]. Beneficial effects of Varenicline and Riluzole have been reported [6–8]. However, according to a consensus paper on management of degenerative CA, these findings need to be further confirmed in further placebo-controlled trials, in particular long-term and disease-specific studies [8, 9]. To date, the only treatment recommendation for CA is physiotherapy [10] and new therapeutic options are needed.

### Acetyl-DL-leucine

Acetyl-DL-leucine (Tanganil; Pierre Fabre, Castres, France) is an acetylated derivative of a natural essential amino acid. Although it has been used for more than 50 years, mainly in France for the symptomatic treatment of acute vertigo and dizziness and improvement of central vestibular compensation [11], the therapeutic mode of action of acetyl-DL-leucine has so far not been very well examined. It may act due to its direct effect on neurons, as was shown in the vestibular nuclei [11]. In vitro studies in guinea pigs demonstrated that acetyl-DL-leucine acts mainly on abnormally hyperpolarized and/or depolarized vestibular neurons by normalizing the membrane potential and has only a minor effect on the membrane potential of vestibular neurons during normal resting potential [12]. In unilateral neurotomy and labyrinthectomy, the agent was described to normalize the vestibular asymmetry with showing an effect seen only in the subgroup of patients with residual vestibular function [11]. Due to the phylogenetical and electrophysiological similarities and close interactions between vestibular and deep cerebellar neurons [13], we had hypothesized that there may also be a positive effect on ataxic symptoms in cerebellar disorders. In an animal model on acute unilateral vestibulopathy, it was found that acetyl-DL-leucine improves compensation of postural symptoms most likely by activation of the vestibulocerebellum, since there was a significant increase of the regional cerebral metabolic rate for glucose in the paraflocculus/flocculus [14]. On cellular levels, it was demonstrated that acetyl-DL-leucine restores the membrane potential of hyperpolarized/depolarized vestibular neurons after unilateral labyrinthectomy in guinea pigs [12]. This mechanism may be mediated by its direct interactions with membrane phospholipids such as phosphatidylinositol 4,5-bisphosphate, which influences ion channel activity [15]. Thereby, acetyl-DL-leucine can stabilize the membrane potential. The input from cerebellar Purkinje cells and mossy/climbing fiber collaterals controls the action potential of the vestibular and the cerebellar nuclei [16], which in turn project to the brainstem, thalamus and spinal cord [13]. Therefore, acetyl-DL-leucine may act through afferent and efferent projections on upstream and downstream structures, thus influencing movement control.

### Trial rationale

In a first case series on 13 patients with different types of hereditary and non-hereditary non-acquired CA, we reported positive effects of acetyl-DL-leucine (5 g per day for one week) on the motor function measured by the Scale for the Assessment and Rating of Ataxia (SARA) and the Spinocerebellar Ataxia Functional Index (SCAFI) [17]. The agent was well tolerated [17]. Mean total SARA decreased from 16.1 ± 7.1 (mean ± SD) at baseline to 12.8 ± 6.8 after one week on medication (*p =* 0.002), and patients showed better performance in the SCAFI consisting of the 8-m-walking-time (8 MW), 9-Hole-Peg-Test of the dominant hand (9HPTD) and the PATA rate task (timed speech task where the patient is asked to repeat “PATA” as quickly and distinctly as possible for 10 s two times). Preliminary FDG-PET-data in a case series of 18 patients suffering from degenerative CA with different etiologies showed central compensation processes in the group of responders mainly in the medulla (vestibular nuclei), midbrain (vestibular integration centers), thalami, basal ganglia and insular regions rather than neurons in the cerebellum, the primary site of dysfunction in CA syndromes [18]. In gait analysis, acetyl-DL-leucine improved the coefficient of variation of stride time in 14 out of 18 cerebellar patients. Acetyl-DL-leucine showed a reduction of gait variability during slow walking [19], whereas aminopyridines have been shown to improve gait variability mainly during fast walking [20, 21]. Subjective ambulatory gait scores and the SARA score also improved under treatment [19]. Further, in a case series on 12 patients with Niemann-Pick type C, a lysosomal storage disorder, there was also a significant improvement of symptoms of ataxia [22]. On the other side, the use of the liquid formula of acetyl-DL-leucine 5 g once daily for 7 days in 10 patients with degenerative CA failed to confirm a treatment benefit of the drug in combination with a short-term physio— and occupational therapy, although 7 out of 10 patients reported subjective improvement [23]. However, all these case studies provide limited evidence and have considerable methodological limitations. Efficacy outcomes were assessed unblinded, rendering objective evaluation of treatment response difficult. Large-scale double-blind randomized controlled trials are highly warranted. Based on these results, we designed the prospective ALCAT trial (Effects of **A**cetyl-DL-**L**eucine on **C**erebellar **AT**axia) to investigate the efficacy and safety of acetyl-DL-leucine with the aim of demonstrating superiority over placebo.

## Methods/Design

### Trial design and setting

The ALCAT trial is an investigator-initiated, multicenter, randomized, double-blind, placebo-controlled, 2-treatment 2-period crossover phase III trial. The study was initiated at the Department of Neurology and the German Center for Vertigo and Balance Disorders (University of Munich) and the Friedrich-Baur-Institute, and has been subsequently extended to other study sites located in Germany (University of Tuebingen, University of Essen, and DZNE Bonn), Austria (Innsbruck) and will be expanded to the Netherlands (Nijmegen). The study has been approved by the responsible ethics committees in Germany (project number 248–15 fed) as well as the ethics committee in Austria (project number AN2015–0252 355/2.1) and has been submitted to the ethics committee in the Netherlands. Furthermore, the study has been approved by the legal medical regulatory authorities (Federal Institute for Drugs and Medical Devices, in German: Bundesinstitut für Arzneimittelsicherheit und Medizinprodukte - BfArM in Germany, Austrian Agency for Health and Food Safety Ltd. AGES, in German: Österreichische Agentur für Gesundheit und Ernährungssicherheit GmbH in Austria, Central Committee on Research Involving Human Subjects, in Dutch: Centrale Commissie Mensgebonden Onderzoek – CCMO in the Netherlands). The first patient was randomized on January 25, 2016. The ALCAT trial is conducted in accordance with the International Conference for Harmonisation (of Technical Requirements for Pharmaceuticals for Human Use) - Good Clinical Practice Guideline (ICH-GCP) and the Declaration of Helsinki. The trial has been prospectively registered at www.clinicaltrialsregister.eu (EudraCT no. 2015–000460–34) and https://www.germanctr.de (DRKS-ID: DRKS00009733) on January 15, 2016. Patient insurance for the study has been arranged (policy number for Germany 39 130537 03026, for Austria 07208763–1, for Netherlands 081 50474–14005).

### Patient population and eligibility criteria

Patients are screened for eligibility according to the inclusion and exclusion criteria. To be eligible for the study, patients aged 18 years or older must present with the clinical symptom of ataxia (hereditary or non-hereditary, non-acquired CA) with at least 3 points in the SARA total score and be able to understand and follow instructions and to give informed consent. All relevant medical and non-medical conditions should be taken into consideration when deciding whether this protocol is suitable for a particular subject. The exclusion criteria were chosen according to available data on acetyl-DL-leucine from the French Agence nationale de sécurité du médicament et des produits de santé (http://agence-prd.ansm.sante.fr/php/ecodex/notice/N0126720.htm, accessed on 24.04.2015, dated on 20.02.2007) and included, in particular, hypersensitivity to the agent. For a detailed description of inclusion and exclusion criteria see Table 1.

**Table 1:** Inclusion and exclusion criteria for patient selection

Inclusion criteria	Exclusion criteria
• Clinically confirmed CA with a SARA total score ≥ 3 (range 0-40) (CA (hereditary or non-hereditary, non-acquired) • Patient did not receive any of the following prohibited medication within 4 weeks prior to randomization: o Aminopyridines o Acetyl-DL-leucine o Riluzole o Gabapentin o Varenicline o Chlorzoxazone • The ability to follow study instructions and likely to attend and complete all required visits • Written informed consent of the subject prior to any study-specific intervention • Age ≥ 18 years	• Subject is not able to give consent • Onset of ataxia in association with stroke, encephalitis, sepsis, hyperthermia or heat stroke • Toxic causes for ataxia of cerebellar type • Rapid progression of ataxia (development of severe ataxia in less than 12 weeks) • Subject suffers from any of the following: o chronic diarrhea o unexplained visual loss o malignancies o insulin-dependent diabetes mellitus • Ataxia due to multiple sclerosis, ischemia, hemorrhage or tumor of the posterior fossa as confirmed by imaging • Ataxia due to clinically likely multisystem atrophy type C (MSA-C) • Diagnosis of clinically likely Friedreich’s ataxia • Known history of hypersensitivity to the investigational drug or derivatives • Liver failure defined as AST/ALT > 300 U/l • Simultaneous participation in another clinical trial or participation in any clinical trial involving administration of an investigational medical product within 30 days prior to the beginning of the clinical trial • Subjects with a physical or psychiatric condition which, in the opinion of the investigator, may put the subject at risk, may confound the trial results, or may interfere with the subject’s participation in this clinical trial • Known or persistent abuse of medication, drugs or alcohol • Females of childbearing potential, who are not using and not willing to use medically reliable methods of contraception for the entire study duration as listed in the patient informed consent form • Current or planned pregnancy or nursing women • Patient has received any of the following prohibited medication within 4 weeks prior to randomization o Aminopyridines (including sustained-release form) o Acetyl-DL-leucine o Riluzole o Gabapentin o Varenicline o Chlorzoxazone

### Recruitment and patient involvement

Patients will be recruited via personal correspondence and by routine care appointments at specialized tertiary referral centres (Neurological departments of University hospitals). In Germany, we also cooperate with the Deutsche Heredo-Ataxie Gesellschaft e.V. (DHAG), a self-help group of ataxia patients. All eligible CA patients who agree to participate in the study will be provided with a full verbal explanation of the trial and the Patient Information Sheet. This will include detailed information about the rationale, design and personal implications of the study. After information is provided to patients, they will have sufficient time to consider participation before they are asked whether they would be willing to take part in the trial. It is imperative that written consent be obtained before any trial-specific procedures commence.

### Randomization, concealment, and blinding

Participants fulfilling the entry criteria at screening will be randomly assigned in a ratio of 1:1 to receive one of the two treatment sequences (active treatment followed by placebo, or vice versa). The randomization technique is based on permutated balanced blocks with random block length. The procedure considers stratification by study site and by genetic vs. sporadic CA to ensure balanced strata, maintaining allocation concealment. The allocation sequence will be generated by an independent person from the Institute of Medical Informatics, Biometry and Epidemiology (IBE) of the University of Munich who is not involved in assessing study outcomes. Neither the investigators nor other trial staff (data analysts, statisticians) or the patients will be informed about the treatment sequences to which a patient is allocated, and neither has access to the randomization list. Thus, randomisation will be conducted without any influence of the investigators or trial staff. The IBE will provide an internet-based, password-protected randomization tool (“Randoulette”: https://wwwapp.ibe.med.uni-muenchen.de/randoulette), which chooses the treatment sequence for a new patient who fulfills the eligibility criteria and has signed the informed consent. Randoulette will register the patient by his or her screening number, gender, year of birth and strata before the allocated package number is provided. The current trial is subject— and investigator-blinded. Patients, clinicians, core laboratories, and trial staff (including data analysts and statisticians) will be unaware of the treatment that the participant will take during both double blind treatment periods.

Un-blinding will occur in the event of a clinical emergency in which the knowledge of the medication being taken is essential for the participant’s clinical management. The investigator is supplied with an opaque sealed envelope for each participant that contains the corresponding treatment sequence for unblinding. Alternatively, the randomization code can be broken using Randoulette.

### Trial procedures and interventions

**Fig. 1:**
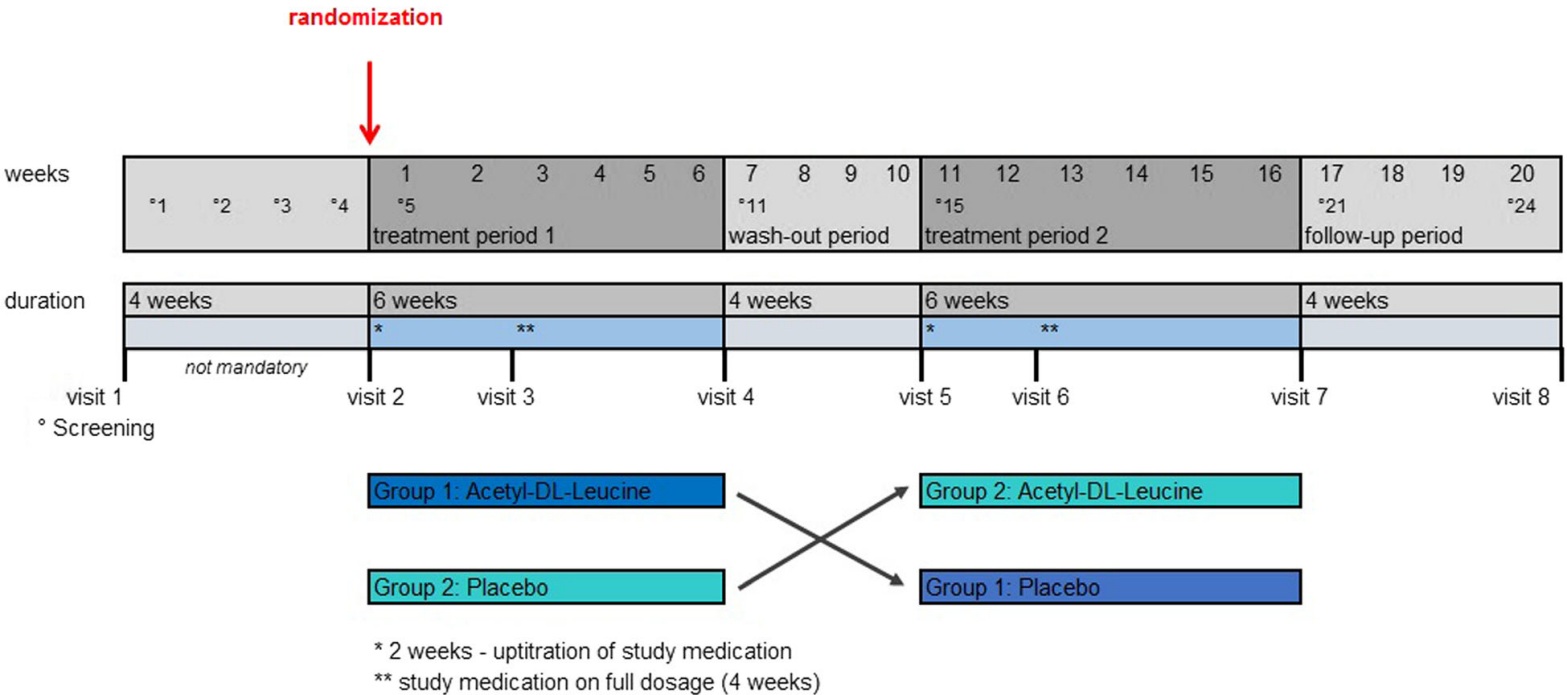
ALCAT trial: crossover intervention scheme. Screening (visit 1) and visit 2 can take place on the same date, if patients are not on prohibited medication within 4 weeks prior to enrolment. If patients have received any of the prohibited medications 4 weeks prior to enrolment, a wash-out period of 4 weeks prior to visit 2 (randomization) is required to rule out a carryover effect

Each treatment sequence consists of a first treatment period of 6 weeks (42 days), followed by a 4-week (28 days) wash-out period, and a second treatment period of 6 weeks (42 days). Finally, a post-treatment follow-up is scheduled 4 weeks (28 days) after the end of the second treatment period (see fig. 1). The 4-week wash-out period between consecutive treatments is assumed to be long enough to allow the effects of a treatment to wear off and prevent carry over from one treatment period to the next.

Patients will be screened and assessed for eligibility at visit 1. If within 4 weeks prior to visit 1 a patient has received any of the prohibited medications defined in the eligibility criteria, irrespective of the preceding treatment duration, a wash-out period of 4 weeks (screening period), prior to enrolment is required. Patients eligible for entry in the study will be randomized and assigned to one of the two sequence groups at visit 2. Visits 1 and 2 coincide if patients are not on prohibited medication 4 weeks prior to recruitment. At baseline, data on demographic and clinical characteristics including neurological assessment, ataxia rating scales, information about preceding physio– and speech therapy as well as patient questionnaires will be obtained. Figure 1 displays the crossover intervention scheme. Table 2 lists the schedule of enrolment and assessments together with pre-planned time points for clinic visits. Including the eligibility screening visit 1 and the post-treatment follow-up visit 8, a total of eight study visits (three in each treatment period) are scheduled.

**Table 2:** Schedule of enrolment, interventions, and assessments

	Enrollment		Treatment period 1				Treatment period 2			Close-out
	Before	Eligibility Screening Visit 1^c^	Visit 2 (Baseline)	Visit 3 2 weeks after V2	Visit 4 6 weeks after V2	Wash-out 4 weeks	Visit 5	Visit 6 2 weeks after V5	Visit 7 6 weeks after V5	Follow-up Visit 8 4 weeks after V7
Timeline (days)		0/-28	0	14	42	70	70	84	112	140
Informed consent^a^	X									
Inclusion/exclusion criteria		X								
Medical history, including demographics & medications		X								
Documentation of physiotherapy/speech therapy		X	X^b^	X	X		X	X	X	X
Neurological examination		X								
Blood tests		X^d^			X^d^		X^d^		X^d^	
Randomization			X							
Dispensing of trial drug			X				X			
Return of trial drug					X				X	
Compliance check				X	X			X	X	
Patient questionnaires (EQ-5D-5 L, BDI-II, FSS)			X	X	X		X	X	X	X
Ataxia rating scale: SARA		X	X^b^	X	X		X	X	X	X
Ataxia rating scale: SCAFI			X	X	X		X	X	X	X
Documentation of (S)AEs		X	X^b^	X	X		X	X	X	X
Documentation of concomitant medication (drug history)			X	X	X		X	X	X	X

A delay of -3 and +5 days is acceptable for visits 2 and 5, for all other visits a delay of ± 5 days is acceptable

^a^prior to first study-specific intervention and allocation

^b^If visit 1 and visit 2 take place at the same time, the ataxia rating scale (SARA), documentation of physiotherapy/speech therapy and documentation of (S)AE are assessed only once

^c^If patients are on medication due to cerebellar symptoms at visit 1, a 4-week wash-out (screening period) prior to randomization at visit 2 is required (adherence will again be checked before randomization at V2). Otherwise, visit 1 and visit 2 coincide

^d^incl. negative pregnancy test for women of childbearing potential

### Investigative drug and placebo

In the experimental treatment period, patients will receive tablets of acetyl-DL-leucine 500 mg (Tanganil®, manufactured by Pierre Fabre, Castres, France) (other ingredients: wheat starch, pregelatinized corn starch, calcium carbonate, and magnesium stearate as filling material). Tablets containing the active ingredient will be refilled from original packaging into blisters under sterile conditions and relabeled by the pharmacy of the university hospital of Heidelberg. An identically appearing tablet filled with wheat starch, pregelatinized corn starch, calcium carbonate, and magnesium stearate but not containing any active ingredient will be administered as placebo. Study medication blisters will be packed in boxes and study kits will be delivered to the participating centers.

Study medication will be delivered to the patient at the beginning of a 6-week treatment period (visits 2 and 5, respectively). Patients are instructed to apply a 2-week up-titration scheme and will be provided with written information. The up-titration scheme is as follows: in the first week an initial dosage of 1.5 g per day (500 mg t.i.d.), followed by 3 g per day (500 mg 2 tablets t.i.d.) in the second week. After that (and another study visit), the full dosage of 5 g per day (500 mg 3–3–4) will be administered for another 4 weeks. If adverse events (AEs) are noted, patients are permitted to down-titrate to a minimum dosage of 1.5 g per day (in the maintaining phase from week 3 to 6) within the current treatment period, which is then considered to be their maximal dose. The patients are instructed that the study medication has to be taken at least 30 min before and at least 2 h after a meal. Treatment adherence will be assessed by counting tablets at the end of the treatment period, and by recording the number of skipped intakes.

### Study objectives and outcomes

The primary objective of the ALCAT trial is to demonstrate that acetyl-DL-leucine is effective at improving motor function measured by the SARA total score. The primary efficacy endpoint is the absolute change in the SARA total score from period-level baseline to the end of the 6-week treatment period, i.e. the difference between post-treatment values at the end of each treatment period and the corresponding period-level baseline values:

$$ \mathrm{Delta}\ \left(\mathrm{SARAtotal}\right) = \mathrm{SARAtotal}\ \left(\mathrm{post}\hbox{-} \mathrm{treatment}\right)\ \hbox{--}\ \mathrm{SARAtotal}\ \left(\mathrm{period}\ \mathrm{baseline}\right) $$

We presume a minimum clinically relevant difference in the SARA total score of 1.5 points. The secondary objectives are to determine whether Acetyl-DL-Leucine is effective at:

- improving motor function measured by the Spinocerebellar Ataxia Functional Index (SCAFI) and SARA subscore items
- improving QoL, as well as the common comorbidities depression and fatigue.

The secondary efficacy outcome measures will be absolute changes in the subscores of SARA as well as the SCAFI total score and subscores of SCAFI (8 MW testing gait, 9HPT testing limb ataxia and the PATA rate task for speech). Furthermore, patient-reported outcomes using the EuroQoL 5 dimensions and 5 level version (EQ-5D-5 L), Beck’s Depression Inventory (BDI-II) as well as the Fatigue Severity Scale (FSS) will be recorded.

For all these scores, the treatment effect will be assessed at the end of each treatment period and at the post-treatment follow-up visit.

During the whole study period any AE (any untoward medical occurrence, including an abnormal laboratory finding, regardless of its causal relation to the study treatment) will be recorded.

#### Ataxia rating scales

The clinical severity of ataxia will be assessed by two different clinical ataxia rating scales — namely SARA [24] and SCAFI [25, 26]. The scores will be assessed by the investigators. Both scales assess ataxia and dysarthria, and are widely used in clinical practice [26] and clinical trials covering the whole range of impaired motor function in ataxic patients. The scales are measured at each clinic visit.

The SARA total score serves as a key inclusion criterion measuring the severity of ataxia prior to enrollment. The SARA total score has eight categories reflecting neurologic manifestations of CA. Each category represents a crucialmovement feature in CA rated by an examiner (gait, stance, sitting, speech disturbance, finger chase, nose-finger test, fast alternating hand movements, heel-shin slide) resulting in a score ranging from 0 (no ataxia) to 40 points (most severe ataxia). It is a reliable and valid clinical scale with a high internal consistency that measures the severity of ataxia and increases with disease stage [25, 27, 28].

The SCAFI includes the 8 MW testing gait, the 9HPT testing limb ataxia and the PATA rate task for speech and therefore represents vital movement features. In contrast to the SARA, the SCAFI uses a timed approach rated by an examiner. After the assessment, raw scores are transformed into reciprocals and converted into subtest Z-scores. The resulting SCAFI is defined as the arithmetic mean of all three Z-scores.

#### Patient questionnaires for quality of life, depression and fatigue

A self-administered questionnaire to evaluate QoL, namely the EQ-5D-5 L [29] (multiple choice questionnaire and a visual analogue scale) will be used. The EQ-5D-5 L is a standardized measure of health status that provides a simple, generic measure of health for clinical and economic appraisal and consists of 2 parts — the EQ-5D descriptive system and the EQ visual analogue scale (EQ-VAS). The EQ-5D-5 L descriptive system comprises the following 5 dimensions: mobility, self-care, usual activities, pain/discomfort and anxiety/depression. Each dimension has 5 levels: no problems, slight problems, moderate problems, severe problems, and extreme problems.

As depression and fatigue are known common comorbidities in patients with CA, trial participants are instructed to complete the BDI-II [30–32] and FSS [32] questionnaires at each clinic visit. The BDI-II is a multiple choice self-report inventory for measuring the severity of depression and is composed of items related to symptoms of depression such as hopelessness and irritability, emotions such as guilt or feelings of being punished, as well as physical symptoms such as fatigue, weight loss and lack of interest in sex. The FSS captures the patient’s experience of mental or psychological fatigue and how it interferes with performing certain activities (exercise, work and family life). It is a self-reporting scale of 9 items. The mean of all answered items represents the fatigue severity.

#### Laboratory examinations

A routine blood sample will be taken to exclude liver or kidney failure, and a pregnancy test for women of childbearing potential will be performed. A pregnancy test is not required for postmenopausal (amenorrhea >12 months), surgically sterilized or hysterectomized women. The following laboratory parameters will be assessed (but not routinely documented in the CRF): sodium, potassium, creatinine, serum bilirubin level, AST, ALT, urea, ALP, TSH, hemoglobin, erythrocytes, hematocrit, thrombocytes, leukocytes, pregnancy test for women of childbearing potential. In a case of an AE, laboratory parameters will be documented. The total amount of blood taken per subject during the entire trial will be approximately 20 ml. Laboratory examinations are carried out at visit 1, visit 4, visit 5 and visit 7.

### Concomitant drug and non-drug therapies

Trial participants should not begin physiotherapy or speech therapy while they are enrolled in the trial. If they are already under therapy, the amount of sessions of physiotherapy and speech therapy (measured in hours of therapy per week) will be documented in the patient’s medical record and in the case report form (CRF) during the trial and for 6 months prior to randomization. To adhere to the protocol, the non-pharmacological concomitant therapy should be continued with the same intensity while the patient is enrolled in the trial.

Guided by the eligibility criteria, the administration of 4-aminopyridine, Riluzole, Varenicline, Gabapentin or Chlorzoxazone is not allowed during the trial because of a possible beneficial effect in CA.

### Participation discontinuation and follow-up of participants ‘off protocol’

If a participant withdraws from the study, the reason will be documented. ‘Off protocol’ is defined as those study participants who cease trial medications on clinical grounds. Regardless of the decision to continue with study medication, these participants will be asked to participate in all scheduled follow-up appointments, as if they were maintaining full participation, in order to prevent further missing data. Those who are unwilling or unable to do this will be asked to agree to a phone or mail follow-up, and/or permission for study staff to continue ascertainment of outcomes.

### Statistical planning and analyses

#### Power considerations and sample size calculation

The sample size was calculated on the basis of the primary hypothesis, using our own preliminary case series data (13 patients [17]) and a similar placebo-controlled trial (20 patients [7]) investigating the efficacy of varenicline on the change in SARA total score in patients with SCA 3. Hence, assuming a minimum clinically relevant difference in the SARA total score of 1.5 points [25] to be detected (i.e. the absolute change on acetyl-DL-leucine is 1.5 score points better than the change on placebo) and a standard deviation of the individual SARA change of 4.2, a sample size of 86 (85 is calculated but 2 sequences are needed) in total is will have 90% power to detect a difference in means of 1.5, using a paired *t*-test with a 0.05 two-sided significance level [Software used: nQuery Advisor 7.0]. On the basis of our experience with patient compliance in previous studies, we expect a dropout rate of about 20%. Thus, a total of 108 patients have to be enrolled. With a proportion of about 50–55% recruited patients out of the number to be screened, about 200 patients have to be assessed for eligibility. There is no interim analysis planned for this study.

#### Statistical analyses for primary and secondary endpoints

Efficacy analyses are based on the intention to treat (ITT) principle in that all participants randomized will be analyzed according to the treatment sequences to which they were allocated and irrespective of the extent of intervention received. In this study, the term ‘full analysis set’ is used to describe the analysis set, which is as complete as possible and as close as possible to the ITT ideal of including all randomised participants (i.e, the ITT population). All statistical tests are two-sided, and the significance level alpha is set to 5%.

For the primary efficacy outcome change in SARA total score (DeltaSARA), the null hypothesis will be tested that there is no difference in mean change between placebo and acetyl-DL-leucine.

Absolute change, calculated as the difference between the period-level baseline score and the score measured at the 2-week and 6-week visits of the acetyl-DL-leucine/placebo period, will be considered for a model-based primary efficacy analysis. If not revised in the statistical analysis plan (SAP), a linear mixed effects model for DeltaSARA will be performed (fixed effects: factor for treatment, visit, treatment-by-visit interaction, and mean of both period-level baseline SARA total scores as covariate; normally distributed patient-specific random intercepts and slopes) in order to deal with repeated measurements within periods and measurements not made at equivalent times in each subject due to unscheduled or missed clinic visits. This subject-specific modelling approach allows individual change scores over time to be calculated. To analyse the differences between both interventions at the end of the 6-week treatment period, 95% confidence intervals will be provided to quantitatively describe treatment effects.

Sensitivity analyses will be performed on a per protocol approach which takes into account treatment adherence and compliance with the trial protocol. Additional efficacy analyses adjusting for genetic vs. sporadic CA, gender, age, or trial site, will be performed. In addition, the robustness of the overall efficacy result will be investigated by analyses adjusting for the amount of physiotherapy the patient received when he or she was enrolled in the trial (which should be comparable to the amount received before randomization), as considered appropriate during the blinded data review and depending on the data quality. Since this analysis will also be based on variables measured after randomization, the susceptibility for bias will be given consideration and will be discussed.

In case of a statistically significant primary efficacy result, confirmatory testing will be extended to the EQ-5D-5 L (VAS and descriptive scale). SCAFI (total score and 3 subscores), SARA subscale items, as well as patient-reported outcomes BDI-II and FSS are not considered in the hierarchical multiple testing procedures. Descriptive comparisons between treatment groups at the end of the 6-week acetyl-DL-leucine/placebo period will be performed using a two-sided Wilcoxon signed rank test, and additionally, Hodges-Lehmann estimation of the location shift between the two groups will be provided, as considered appropriate based on the blinded data. The same non-parametrical tests will be employed to estimate treatment differences at the follow-up visit 8 (close-out visit). Further methodological details will be provided in the SAP.

#### Safety assessment

Outcome assessors will judge the severity (mild, moderate, or severe), seriousness, and causality (definitely related, probably related, possibly related, possibly not related, definitely not related to the intervention, or not assessable). All adverse events will be listed by trial site and patient and displayed in summary tables. The incidence of adverse events and their relationship to the study drug will be analyzed descriptively, guided by the Medical Dictionary for Regulatory Activities (MedDRA) classification.

### Data collection

Results from the trial assessments will be recorded in an electronic CRF (e-CRF) via a validated open-source electronic data capture (EDC) system based on the OpenClinica® Community Edition. At each clinical site, study personnel will enter data directly into the EDC system. Data will be transferred on a regular basis to the official study database stored in SAS (Version 9.2 for Linux, SAS Institute, Cary, NC) for quality checks and query management. Statistical analyses will be performed using SAS, as well as the software package R version 3.3.0 or higher [33].

### Data safety and monitoring board (DSMB)

An independent DSMB has been established. The function of the DSMB is to monitor the course of the study and if necessary to give a recommendation to the coordinating investigator and sponsor of the trial for discontinuation, modification or continuation of the study. Furthermore, the DSMB will periodically review the safety data of Development Safety Update Reports.

## Discussion

Because there is no approved causal or symptomatic drug therapy yet, there is an urgent need for effective and well-tolerated drug treatment. Based on different case series in hereditary or non-hereditary non-acquired CA of different etiologies [17, 19, 22] and its good safety profile (since 1957 on the market in France for symptomatic dizziness and vertigo treatment) the modified amino-acid acetyl-DL-leucine is a good candidate.

In this multicenter, multinational, randomized, double-blind, placebo-controlled, phase III trial, short-term treatment efficacy and safety are evaluated with a 2-treatment 2-period crossover design comprised of 6-week treatment periods. The primary inclusion criterion SARA total score of at least 3 points ensures that patients are at least moderately affected by the disease and allows CA patients with a wide spectrum of clinical severity to be included. Except for the blood tests, all examinations and assessments applied are non-invasive and also performed in clinical routine. If superiority of acetyl-DL-leucine compared to placebo intervention can be established, this medication could offer a complete new therapeutic approach for the target population. Due to the high prevalence and the participation of many centers in three European countries, recruitment to reach the target sample size of 108 participants seems achievable within the planned time frame of 32 months. Due to the pragmatic nature of the trial design, the primary question of interest relates to intervention effectiveness, i.e. whether the intervention works under real-life conditions. In particular, trial participants are allowed to maintain physio- or speech therapy during the trial provided that they have already started this therapy before recruitment.

Of course, this trial has some limitations: first, so far we do not know what the optimal dosage is. The dose of 5 g per day used in this trial is based on case series. Second, we do not know what the optimal dosage interval is. Third, since the plasma levels are not measured, we cannot correlate them with the clinical effect and we cannot exclude patients taking acetyl-DL-leucine in addition to the study medication. Fourth, as we include multiple types of CA (hereditary or non-hereditary non-acquired) we will not know if the agent is more effective with one CA than with another due to small numbers of the different CA types. Last, but not least, the observational period is quite short, so we cannot evaluate the effect of the drug on the long-term progression of the disease compared to placebo intervention. This, however, could easily be done as soon as its efficacy and safety in symptomatic treatment is demonstrated.

### Trial status

At the time of manuscript submission, the study design had been evaluated by independent international reviewers and has been approved by the responsible ethics committee of the University of Munich and the ethics committee in Austria as well as the authorities in Germany (BfArM) and Austria (AGES). The study is being prepared for submission in the Netherlands. The first study participant was randomized on January 25, 2016 with the opening of the trial site of the coordinating investigator. As of October, 1^st^, we have included a total of 92 patients.

## Abbreviations

(S)AE(s): (serious) adverse event(s)
(S)CA: (spino-) cerebellar ataxia
8 MW: 8-m-walking-time
9HPTD: 9-Hole-Peg-Test of the dominant hand
AGES: Österreichische Agentur für Gesundheit und Ernährungssicherheit GmbH in Austria (Austrian agency for health and food safety Ltd.)
BDI-II: Beck’s depression inventory
BfArM: Bundesinstitut für Arzneimittelsicherheit und Medizinprodukte (Federal institute for drugs and medical devices)
CCMO: Centrale Commissie Mensgebonden Onderzoek (Central committee on research involving human subjects)
CRF: Case report form
DHAG: Deutsche Heredo-Ataxie Gesellschaft e.V.
DSMB: Data safety and monitoring board
e-CRF: Electronic CRF
EDC: Electronic data capture
EQ-5D-5L: EuroQol 5 dimensions and 5 level version
EQ-VAS: EuroQol Visual analogue scale
FSS: Fatigue severity scale
ICH-GCP: International conference for harmonisation (of technical requirements for pharmaceuticals for human use) - good clinical practice guideline
ITT: Intention to treat
MedDRA: Medical dictionary for regulatory activities
MSA-C: Multisystem atrophy type C
QoL: Quality of Life
SAP: Statistical analysis plan
SARA: Scale for the assessment and rating of ataxia
SCAFI: Spinocerebellar ataxia functional index
SD: Standard deviation
SDP: Sponsor’s delegated person

## References

[1] Jacobi H (2011). The natural history of spinocerebellar ataxia type 1, 2, 3, and 6: a 2-year follow-up study. Neurology.

[2] Klockgether T (2012). Sporadic adult-onset ataxia of unknown etiology. Handb Clin Neurol.

[3] Perlman SL (2004). Symptomatic and disease-modifying therapy for the progressive ataxias. Neurologist.

[4] Lopez-Bastida J (2008). Social economic costs and health-related quality of life in patients with degenerative cerebellar ataxia in Spain. Mov Disord.

[5] Ilg W, Bastian AJ, Boesch S, Burciu RG, Celnik P, Claaßen J, Feil K, Kalla R, Miyai I, Nachbauer W, Schöls L, Strupp M, Synofzik M, Teufel J, Timmann D. Consensus paper: management of degenerative cerebellar disorders. Cerebellum. 2014;13(2):248-68. doi:10.1007/s12311-013-0531-6. 10.1007/s12311-013-0531-6 PMC4344126 24222635

[6] Ristori G (2010). Riluzole in cerebellar ataxia: a randomized, double-blind, placebo-controlled pilot trial. Neurology.

[7] Zesiewicz TA (2012). A randomized trial of varenicline (Chantix) for the treatment of spinocerebellar ataxia type 3. Neurology.

[8] Romano S (2015). Riluzole in patients with hereditary cerebellar ataxia: a randomised, double-blind, placebo-controlled trial. Lancet Neurol.

[9] Ilg W (2014). Consensus paper: management of degenerative cerebellar disorders. Cerebellum.

[10] Ilg W (2009). Intensive coordinative training improves motor performance in degenerative cerebellar disease. Neurology.

[11] Ferber-Viart C, Dubreuil C, Vidal PP (2009). Effects of acetyl-DL-leucine in vestibular patients: a clinical study following neurotomy and labyrinthectomy. Audiol Neurootol.

[12] Vibert N, Vidal PP (2001). In vitro effects of acetyl-DL-leucine (tanganil) on central vestibular neurons and vestibulo-ocular networks of the guinea-pig. Eur J Neurosci.

[13] Highstein SM, Holstein GR (2006). The anatomy of the vestibular nuclei. Prog Brain Res.

[14] Gunther L (2015). N-acetyl-L-leucine accelerates vestibular compensation after unilateral labyrinthectomy by action in the cerebellum and thalamus. PLoS One.

[15] Suh BC, Hille B (2008). PIP2 is a necessary cofactor for ion channel function: how and why?. Annu Rev Biophys.

[16] Witter L (2011). The cerebellar nuclei take center stage. Cerebellum.

[17] Strupp M (2013). Effects of acetyl-DL-leucine in patients with cerebellar ataxia: a case series. J Neurol.

[18] Becker-Bense S, Feuerecker R, Xiong G, Feil K, Bartenstein P, Strupp M, Dieterich M (2015). Effects of acetyl-DL-leucine on the cerebral activation pattern in cerebellar ataxia (FDG-PET study) - Oral Sessions No. O1201. Eur J Neurol.

[19] Schniepp R (2016). Acetyl-DL-leucine improves gait variability in patients with cerebellar ataxia-a case series. Cerebellum Ataxias.

[20] Schniepp R (2012). 4-aminopyridine and cerebellar gait: a retrospective case series. J Neurol.

[21] Schniepp R (2011). 4-Aminopyridine improves gait variability in cerebellar ataxia due to CACNA 1A mutation. J Neurol.

[22] Bremova T, M.V., Amraoui Y, Mengel E, Reinke J, Kolníková M, Strupp M Acetyl-DL-leucine in Niemann-Pick type C: a case series. Neurology. Neurology, 2015. In press. 10.1212/WNL.0000000000002041 26400580

[23] Pelz JO (2015). Failure to confirm benefit of acetyl-DL-leucine in degenerative cerebellar ataxia: a case series. J Neurol.

[24] Subramony SH (2007). SARA--a new clinical scale for the assessment and rating of ataxia. Nat Clin Pract Neurol.

[25] Schmitz-Hubsch T (2006). Scale for the assessment and rating of ataxia: development of a new clinical scale. Neurology.

[26] Schmitz-Hubsch T (2008). SCA Functional Index: a useful compound performance measure for spinocerebellar ataxia. Neurology.

[27] Weyer A (2007). Reliability and validity of the scale for the assessment and rating of ataxia: a study in 64 ataxia patients. Mov Disord.

[28] Yabe I (2008). Usefulness of the scale for assessment and rating of ataxia (SARA). J Neurol Sci.

[29] Rabin R, de Charro F (2001). EQ-5D: a measure of health status from the EuroQol Group. Ann Med.

[30] Schmitz-Hubsch T (2010). Self-rated health status in spinocerebellar ataxia--results from a European multicenter study. Mov Disord.

[31] Schmitz-Hubsch T (2011). Depression comorbidity in spinocerebellar ataxia. Mov Disord.

[32] Brusse E (2011). Fatigue in spinocerebellar ataxia: patient self-assessment of an early and disabling symptom. Neurology.

[33] R Core Team, R: A language and environment for statistical computing. R Foundation for Statistical Computing,. Vienna, Austria 2016. http://www.R-project.org/.

